# The Biological Significance of Calmodulin Binding to Lipids

**DOI:** 10.3390/biology15050396

**Published:** 2026-02-28

**Authors:** Danton H. O’Day

**Affiliations:** 1 Department of Biology, University of Toronto Mississauga, Mississauga, ON L5L 1C6, Canada; danton.oday@utoronto.ca; 2 Cell and Systems Biology, University of Toronto, Toronto, ON M5S 3G5, Canada

**Keywords:** calmodulin binding, prenylated lipid binding, Rac1, RalA, KRAS4b, phosphatidylserine, phosphatidylethanolamine, sphingolipids, protein translocation, biomembrane fusion

## Abstract

In addition to binding to and regulating over 400 different proteins, calmodulin (CaM) also binds to lipids. Binding occurs to the prenylated tails of various small GTPases, to specific lipids in biological membranes and to free lipids in the cytoplasm. Here, CaM binding to Rac1, RalA, and KRAS4b is covered, emphasizing its importance in protein translocation from the cell membrane to the cytosol and its resultant impact on cell signaling. Binding phosphatidylserine and phosphatidylethanolamine in membranes not only leads to the tethering of CaM, but also to the disruption of lipid bilayers. Binding to sphingolipids also occurs, an event that acts as a competitive inhibitor of CaM function. The mechanism through which CaM binds to lipids is also examined. In total, the current state of affairs regarding calcium-dependent CaM–lipid binding is reviewed, including potential therapeutic uses, setting the stage for future work on this important biological event.

## Simple Summary

Calmodulin is a small, essential regulatory protein that senses and responds to cellular calcium ion levels. Its unprecedented ability to bind to and control over 100 proteins in the absence of calcium and more than 300 when activated by calcium has been well documented. Unexpectedly, calcium-activated calmodulin also binds to lipids, an event reviewed here for the first time. The binding occurs to the lipid tails of certain prenylated proteins that use the tail to localize to membranes. One effect of this binding is to pull the protein out of the membrane, in turn altering its functions, which is an event linked to multiple forms of cancer and certain cardiovascular diseases. Binding also occurs to individual lipids localized within membranes that can either tether calmodulin to them or disrupt membrane integrity. Lipid binding can also cause calmodulin to dislodge from binding proteins, thus inhibiting calmodulin’s regulatory functions. Calmodulin–lipid binding employs the identical binding region as calcium-mediated protein binding, potentially offering new options for therapeutic development. The multiple functions of calmodulin–lipid binding add further insight into the critical cellular and biomedical functions of this primary calcium sensor and effector.

## 1. Introduction

**Figure 1:**
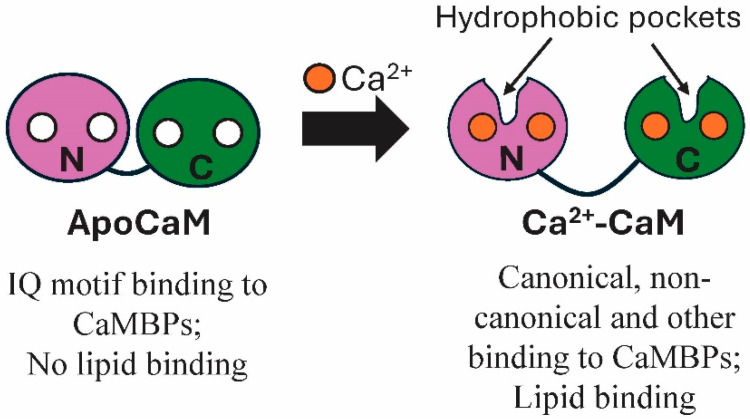
Calcium-mediated transformation of condensed apoCaM into extended Ca^2+^–CaM. ApoCaM binds proteins typically via IQ motifs but does not bind to lipids. Calcium binding opens hydrophobic pockets in Ca^2+^–CaM for CaMBP binding via canonical, non-canonical and other binding motifs. Ca^2+^–CaM can also bind to lipids via its hydrophobic pockets and other modes of interaction as detailed in the body of the text.

The number of biological events that are under control of the calcium sensor and effector calmodulin (CaM) is unparalleled by any other regulatory protein and has been well reviewed [1,2,3,4]. A comparatively small (152aa, 16.7 kDa) bi-lobed Ca^2+^-binding protein, CaM, binds target binding proteins (CaMBPs) in a Ca^2+^-free (apoCaM) state or when bound to Ca^2+^ (Ca^2+–^CaM) (Figure 1). ApoCaM binding typically involves IQ motifs [5]. Calcium-binding leads to a significant conformational change, transforming condensed apoCaM’s ability to bind one select group of 100+ proteins to activated dumbbell-shaped Ca^2+^–CaM that can bind to over 300 different proteins. Calcium-dependent binding to CaMBPs typically involves hydrophobic amino acid binding domains in canonical and non-canonical sequences, but other modes of interaction also occur [1,2,3,4]. Operating directly or through its CaMBPs, CaM plays central roles in autophagy, cell division, muscle contraction, endocytosis, exocytosis, intracellular transport, gene regulation, metabolic homeostasis, cell death, and neuronal functions, among others. As might be expected, it also operates in a diversity of diseases from cancer to neurodegeneration [6,7].

Ca^2+^–CaM’s dumbbell shape is defined by two almost identical calcium-binding lobes separated by a flexible linker [3]. Of relevance here, calcium-binding opens hydrophobic pockets in each CaM lobe that bind to CaMBPs via an FLMM (Phe, Leu, Met, Met) binding sequence [8,9]. Calcium levels affect the opening of the hydrophobic pockets in each lobe since the C-term lobe (C-lobe) has an approximately 10-fold higher affinity for the cation than the N-term lobe (N-lobe) [10]. This difference allows for sequential binding of the lobes to target proteins, an event that is important in CaM–lipid interactions as detailed below.

An unexpected shift in thinking was required when it was revealed that Ca^2+^–CaM not only binds and regulates a wide diversity of proteins but also binds to lipids. The lipid profile of cells and their diverse functions are beyond this review [11]. Lipids not only exist as components of membranes (e.g., phosphatidylserine, PS; phosphatidylethanolamine, PE; phosphatidylcholine, PC) and bounds to proteins (prenyl groups, myristoyl groups), but also independently as metabolic fuels and precursors (e.g., palmitic acids, oleic acid), as well as signaling molecules (e.g., sphingosylphosphorylcholine, SPC; diacylglycerol, DAG; phosphatidic acid). CaM binds to both free, membrane-associated and protein-bound lipids, but not all lipids can bind to the calcium sensor and effector.

The earliest revelation that lipids could bind to CaM came from studies on certain enzymes. Research on the eukaryotic microbe *Dictyostelium* indicated that calcineurin, the sole CaM-regulated phosphatase, was also activated by arachidonic acid, linoleic acid, and oleic acid [12]. However, subsequent verification of the stimulation of this phosphatase by long-chain unsaturated fatty acids has not been reported. On the other hand, several sphingolipids, including sphingosine, galactosylsphingosine and glucosylsphingosine, have an inhibitory effect on calcineurin, but that effect is due to their direct binding to CaM, not binding to the target enzyme, inhibiting CaM’s availability as an enzyme activator, as discussed below [13].

Continued research began to validate the binding interaction between CaM and lipids. While it is in its research infancy, this binding interaction has significant biological implications that have not been thoroughly assessed. This review sequentially covers the following topics: CaM binding to prenylated proteins, to lipids in biological membranes and to free lipids. It also addresses the mechanism of CaM–lipid binding. Throughout the article, the biological significance of binding is addressed with examples that are summarized in the Discussion. Concluding remarks offer suggestions about its potential therapeutic value and its biological importance in biomembrane fusion events.

## 2. Protein Myristoylation and Calmodulin Binding

As the name reveals, post-translational modifications (PTMs) are changes made to proteins after their synthesis [14]. Over 650 PTMs have been identified, but less than two dozen have been well studied, including phosphorylation, glycosylation, acetylation, methylation, ubiquitination and lipidation, to name a few [15]. PTMs alter protein structure and function, leading to their altered localization, stability and interactions with other cellular components. PTMs involving lipidation, the covalent addition of lipids to proteins, include cholesterylation, myristoylation, palmitoylation, prenylation, and the addition of glycosylphosphatidylinositol (GPI) anchors [16]. Protein lipidation mediates a diversity of events including, but not limited to, protein localization, trafficking, signal transduction and membrane modifications [16,17]. Despite the significant role CaM plays in regulating events mediated by protein lipidation, recent major reviews have failed to cover this topic [16,17]. In addition, a review of “Dolphin”, a comprehensive database documenting protein–lipid interactions, does not mention lipid–CaM binding, emphasizing the need to bring this area to light [18].

There is no evidence that CaM binds directly to cholesterol, palmitoyl groups, or GPI anchors, but there is proof of myristoyl– and prenyl–lipid binding. Nef is a myristoylated protein that is expressed early in human immunodeficiency virus (HIV-1, HIV-2) infections [19]. Studies using NIH3T3 cells revealed Nef binds CaM, an event that is greatly increased in the presence of calcium [20]. N-term binding of Nef does not involve a protein-based binding motif but requires myristoyl group binding to the hydrophobic pocket in CaM lobes, an event that mediates Nef membrane localization. Cellular regulation of Nef–CaM binding has not been detailed to date.

The brain-specific protein kinase C substrate CAP-23/NAP-22 (also CAP23 or BASP1) is involved in neuronal signal transduction and axon regeneration [21]. Lacking a canonical CaMBD, it binds CaM with high affinity via an N-terminal myristoyl group [22]. Myristoyl binding is essential for membrane localization. CaM binding involves hydrophobic pockets in its N- and C-terminal domains, but the role of this binding in overseeing the localization and functions of CAP-23/NAP-22 remains to be studied.

## 3. Protein Prenylation and Calmodulin Binding

While CaM binding to myristoylated proteins requires more analysis, its significance in prenylated protein function is more extensive. Prenylation involves enzymatic attachment of a farnesyl or geranylgeranyl group to a cysteine residue in a protein [23]. The conjugation alters function by mediating the location of proteins to membranes, by affecting their interaction with other proteins and by anchoring them to specific subcellular locales. PRENbase is a specialized database for searching for prenylated proteins (https://mendel.imp.ac.at/PrePS/PRENbase/; accessed on 12 December 2025, 2:30 p.m.) [23]. While there appear to be well over 600 prenylated proteins, only a small group have been well characterized. Prenylated proteins, including small GTPases, nuclear lamins, Gg isoforms of G proteins and certain phosphatases, play critical roles in cell signaling, membrane anchoring and trafficking. Phosphatase of regenerating liver family members are small enzymes involved in cell growth, movement and survival, using a C-term farnesyl group to anchor them to cell membranes [24]. Nuclear lamins use farnesyl groups to tether them to the inner nuclear membrane where they function in the structure and function of the nucleus [25]. The beta subunit of heterotrimeric G proteins binds CaM, and its prenylation is linked to it membrane trafficking [26,27]. There is no evidence CaM binds to lipid PTMs of any of those proteins. However, the role of prenylation and CaM binding in the Ras superfamily of small GTPases is important.

The most mutated oncogene in cancer, Ras, is responsible for approximately 20% of human cancers [28]. The Ras family comprises more than 160 related small G proteins that mediate numerous cellular functions including cytoskeletal dynamics, signal transduction, vesicle trafficking, and nuclear translocations [29]. They do this by switching between an active GTP-bound form and an inactive GDP-bound state, using its dephosphorylation to interact with downstream effector proteins to drive specific events [30]. There are five main family members: Ras, Rho, Rab, Arf (ADP-ribosylation factor), and Ran. Ran proteins lack prenylation sites, but other members typically require prenylation to anchor them to specific membrane locales [31].

Prenylation occurs at a C-terminal CAAX motif (C, cysteine; A, aliphatic amino acid; X, any amino acid) [28]. CAAX box prenylation involves three steps: polyisoprenylation, proteolysis and carboxyl methylation [32]. In the first step, a prenyltransferase attaches a farnesyl (via farnesyltransferase activity) or geranylgeranyl (via geranylgeranyltransferase type I) group. Step one is followed by insertion of the prenylated protein into the membrane. Proteolysis of the CAAX residue to -AAX coupled with additional, limited endoproteolysis and methylation increases the hydrophobicity of the prenylated PTM.

CaM has been shown to interact with a number of prenylated Ras family members. While Arf is prenylated, CaM binding to Arf has not been shown. The regulation by CaM binding to the prenyl group in other family members has only been sufficiently studied for a limited number of small GTPases. As covered here, the role of CaM binding and regulation focusses on certain family members, Rho (Rac1), Ras (KRAS4b), and Rab (RalA, RalB).

## 4. Rac1 Calmodulin Regulation

Rac1 is involved in actin function and the translocation of STAT transcription factors as related to their involvement in promoting cell division and cell motility [33,34]. In keeping with this, Rac1 is upregulated in breast, colon and lung tumors [35]. The prenylation of Rac1 GTPase allows it to bind to lipid bilayers [36]. The functional cycling of membrane-bound Rac1 between its inactive GDP-bound and active GTP-bound state is regulated by GEF (guanine nculeotide exchange factor) that activates it by exchanging GTP for GDP, and by GAP (GTPase activating proteins) that induces GTP hydrolysis to shut down signaling [34]. In the cell’s resting state, Rac1 exists in a complex with RhoGDI, causing its inactivation and sequestering it to the cytoplasm [37]. Phosphorylation of either protein or interaction with other factors leads to the dissociation of the complex, allowing Rac1 to translocate to the membrane where its prenylated tail inserts into the bilayer, anchoring it there. Rac1 then becomes active through the association with its downstream effectors. The bottom line is that the RhoGDI/Rac1 cycle mediates the availability of Rac1 for insertion into membranes via its prenylated tail.

These events are regulated in part by Ca^2+^–CaM binding to and activating Rac1 [38,39]. Research on platelets, HeLa and CHRF-288-11 cells has revealed that CaM plays an essential role in the activation of Rac1—an event that, in turn, drives the reorganization of the cytoskeleton and, in the case of HeLa cells, EGF-induced cell migration [39]. CaM binding to Rac1 involves both a C-term sequence of hydrophobic amino acids and the geranylgeranyl group, neither of which is sufficient to regulate Rac1 independently [38,39,40]. CaM binding to the geranylgeranyl tail of Rac1 does not affect membrane binding, ruling out its role in this event [41]. Considering the diversity of functions mediated by Rac1, it is likely that various regulatory mechanisms come into play, some of which involve CaM binding to the prenyl group and a polybasic region, while others require its prenyl-group, membrane-linked GTPase cycle function. Despite this extensive information, it is interesting that a recent review of Rac1 function fails to mention the role of CaM binding [42].

## 5. KRAS4b Regulation by Lipids and Calmodulin

Of the *Ras* genes, three KRAS oncogenes, KRAS, HRAS, NRAS, are the most mutated cancer genes, with the KRAS4a and KRAS4b protein isoforms being well studied [28,43,44]. KRAS4b is the most actively studied because of its high expression levels in normal and cancer cells. A small oncogenic GTPase, KRAS4b is involved in signal transduction pathways that mediate cell differentiation, proliferation, and survival. The cycling of Kras4b shares events with other small membrane-bound GTPases, since it is regulated by GEFs and GAPs. A crucial GEF linked to cancer that activates it is SOS1 (Son of Sevenless 1), while NF1 (Neurofibromin) is an example of a GAP that regulates the enzyme [41,44]. Prenylation of its C terminus and subsequent recruitment to the plasma membrane are essential to its activity and its regulatory role in activating downstream proteins (e.g., RAF kinases). Studies on HeLa and HEK293 cells revealed that, in response to Ca^2+^ influx, KRAS4b reversibly translocates from the plasma membrane to the cytoplasm, an event that results in enzyme inactivation. The translocation is Ca^2+^–CaM-dependent, involving CaM binding via a farnesyl group on the enzyme [45,46,47].

**Figure 2:**
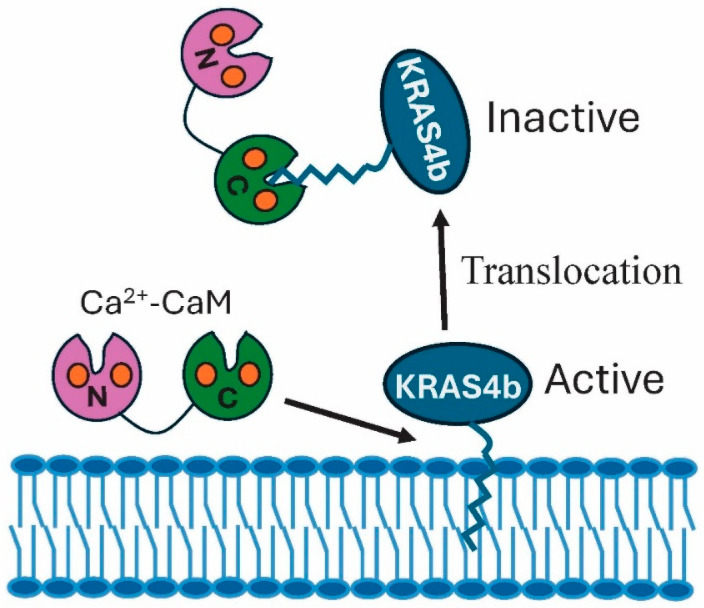
Active KRAS4b is tethered to the plasma membrane via a farnesyl lipid moiety. The hydrophobic pocket in the C-lobe of Ca^2+^–CaM binds to the farnesyl group, releasing KRAS4b from the membrane.

In association with the membrane, the small GTPase recruits different effectors, including RAF and PI3K [48]. Together, various protein combinations activate downstream cancer pathways such as MAPK, PI3K/AKT/mTOR to mediate cell proliferation. The calmodulin-mediated dislodgement of KRAS4b from artificial membranes was assayed using nanodisc biolayer interferometry assay [47]. The data showed the off-rate of the farnesylated protein increased with CaM concentration. At low calcium levels, active KRAS4b binds to biological membranes via its farnesyl group (Figure 2). In response to increased levels of calcium, hydrophobic pockets in each of the CaM lobes open. The prenyl lipid moiety of KRAS4b inserts into a C-lobe hydrophobic pocket, pulling the GTPase out of the membrane, in turn leading to its inactivation [46]. As calcium levels decrease, the hydrophobic pockets close and apoCaM can no longer bind to KRAS4b, allowing it to reinsert into the membrane and become active. In this state, it can associate with effectors that mediate its membrane-associated functions.

## 6. RalA, Canonical and Lipid Calmodulin Binding

As members of the Ras superfamily, RalA and RalB are small GTPases with critical but different roles in cancer. Similarly to other GTPases, they act as molecular switches, alternating between an active GTP-bound and inactive GDP-bound state [49]. RalA activation follows the small GTPase regulatory cycle, being triggered by GEFs, in this case exemplified by RalGEF that exchanges GDP for GTP, and by GAPs (e.g., RalGAP1 complex) that catalyze the hydrolysis of GTP to GDP [49]. As such, they control a diversity of critical cell functions including cytokinesis, filopodia formation, mitochondrial fission and vesicle trafficking, to name a few. RalA and RalB each possess two calmodulin binding domains. A calcium-independent domain exists near the N-term while a calcium-dependent domain resides in the C-term [50]. Since RalA–CaM binding has been studied more, it will be the focus of this section.

RalA serves as a switch between vesicle trafficking and gene regulation. Membrane-bound RalA uses its GTP-mediated small G proteins cycle activity to control vesicular trafficking and exocytosis by tethering GLUT4 to the membrane [51]. When released into the cytoplasm, RalA has other functions, such as sensing cell density to regulate gene expression via mTORC1 [52]. The translocation of RalA from the membrane to the cytoplasm is mediated by Ca^2+^–CaM.

**Figure 3:**
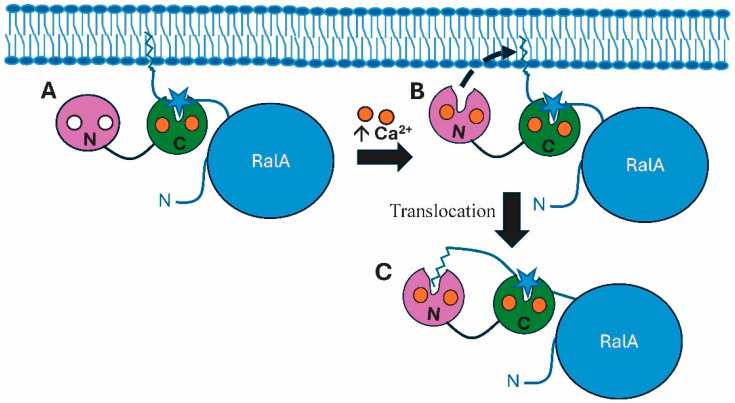
Calmodulin regulation of RalA via geranylgeranyl group binding. (**A**) The C-lobe of calmodulin (CaM) binds to a CaM binding domain (⋆) in the C-terminal segment of RalA. (**B**) The calcium-free N-lobe (white circles) remains unbound until an influx of calcium ions charges it (orange circles). It can then bind to the geranylgeranyl group of RalA that is anchoring it to the membrane. (**C**) Binding of the N-lobe to the geranylgeranyl group dislodges it, allowing the translocation of RalA from the membrane into the cytoplasm. Since increasing calcium levels leads to the binding of membrane-bound prenyl groups attached to proteins (e.g., KRAS4b), this could explain how calcium charging of the N-lobe of CaM would allow binding to the prenyl group of RalA, leading to dislodgement of the protein from the membrane. This figure is adapted from Figure 4 of Chamberlain et al. [53].

The binding between RalA and Ca^2+^–CaM involves both a canonical 1-5-10 calcium-dependent binding domain and a non-traditional binding involving a geranylgeranyl group that anchors the protein to the plasma membrane [53]. Charged with calcium, the C-lobe of CaM binds to the hydrophobic CaMBD in the C-term of RalA (Figure 3). At this point, the C-term geranylgeranyl tail of RalA, becomes imbedded in the membrane. As introduced earlier, CaM undergoes differential binding of calcium in which the C-lobe has a much higher affinity for cation binding than the N-lobe [10]. As calcium levels increase, the N-lobe of CaM also becomes charged with calcium ions, allowing it to bind to the prenyl group. This binding event dislodges RalA from the membrane, causing its translocation into the cytoplasm. Being dislodged from the plasma membrane by CaM inactivates RalA’s membrane functions but initiates its cytoplasmic roles in downstream signaling.

## 7. Calmodulin Binding to Membrane Lipids

Membrane fluidity is a primary event essential for cell movement, cytokinesis, and changes in cell shape. It is also needed for cell fusion events, including receptor-mediated endocytosis, exocytosis, and cell fusion that occurs during fertilization and embryonic muscle formation. These are just a few basic biological processes that depend on membrane fluidity. Experiments with regenerating liver cells revealed that an increased expression of CaM caused a transient decrease in membrane fluidity [54,55]. Live-cell imaging of HeLa cells treated with ionomycin, an ionophore that increases intracellular calcium levels, showed that CaM levels increased in the membrane protrusions induced by these treatments [56]. This demonstrated that CaM makes associations with membranes in response to calcium levels, but it did not reveal whether this association was due to lipid bilayer binding or via binding to membrane-bound CaMBPs. However, research with model lipid membranes showed direct binding to lipids does occur.

The lipid bilayers of eukaryotic membranes comprise over 1000 different types of lipid molecules that vary in their amounts and localization in the plasma membrane, endoplasmic reticulum, Golgi, lysosomes and the various endosomes [57,58]. They are usually grouped as glycerophospholipids, sphingolipids (SLs), and sterols. Within those groups, the main glycerophospholipids include phosphatidylcholine (PC), phosphatidylethanolamine (PE), phosphatidylserine (PS), cardiolipin (CL), phosphatidic acid (PA), and phosphatidylinositol (PI), while major SLs are sphingomyelin and glycosphingolipids. Of interest here are PE, PS, and sphingolipids, because they have been shown to bind to CaM.

PE and PS are both found in the plasma membrane. They predominantly localize to the inner leaflet of the lipid bilayer, an asymmetry maintained by flippases [59]. Both phospholipids are vital, but levels of PS are lower than PE, which comprises around 15–20% of membrane lipid content. On the other hand, the distribution of PE (15–30%) and PS (~7%) between the inner and outer leaflets of endoplasmic reticulum (ER) membranes are almost equivalent [57,60]. Membranes in the trans-Golgi network have highly asymmetric lipid distribution that is similar to PM, with PS and PE accumulated in the inner leaflet at the cytoplasmic side, while early endosomes have a PS and PE distribution that resembles the plasma membrane [57].

Thus, CaM binding to these two phospholipids has the potential to affect the structure and operations of the plasma membrane and a diversity of internal membranes. Using model lipid membranes, Scollo and colleagues experimentally analyzed the interaction between CaM and the membrane phospholipids that comprised them [56]. Their results supported and extended earlier work, revealing that CaM binds to phospholipids, especially PE and PS. They proposed this occurs via a two-stage process that begins with adsorption of Ca^2+^–CaM to PE/PS membranes via transient but essential electrostatic interactions that neutralize the negative charge of the membrane. Hydrogen bonding between Ca^2+^–CaM and PE destabilizes the lipid bilayer, exposing lipid tails that can then bind to the hydrophobic binding pocket of the appropriate Ca^2+^–CaM lobe. One effect of this is to alter membrane fluidity [54,55].

In addition to affecting membrane integrity, since Ca^2+^–CaM binds directly to membrane lipids and since it also forms hydrogen bonds with PE, this suggests that it is a form of tethering of the regulatory protein [56]. Membrane–lipid tethering would complement CaMBP tethering as a means of localizing CaM to the plasma membrane, as exemplified by its association with various ion channels and SNAREs [61,62]. The significance of lipid–CaM tethering and whether it is a stable or transient mode of membrane localization and CaM regulation remains to be studied.

## 8. Sphingolipid Binding to Calmodulin

CaM binds directly to the signaling sphingolipid SPC [63,64]. SPC is both an extracellular and intracellular signaling molecule that affects cardiovascular function, cell proliferation and inflammation via its effects on receptors and its activation of downstream signaling pathways [65]. Research has shown that this sphingolipid acts as both a competitive inhibitor as well as a potential receptor for CaM. A diversity of techniques (e.g., crystallographic, kinetic, thermodynamic, and spectroscopic analyses) confirmed that sphingolipids fit into the conventional hydrophobic pocket on Ca^2+^–CaM, the region historically recognized as the protein-binding domain [63]. Since SPC binding occurs via the calcium-dependent hydrophobic binding pocket of Ca^2+^–CaM, this blocks the pocket, preventing binding to and activation of CaMBPs [64]. It may also displace pre-bound CaMBPs. As a competitive inhibitor of CaM, SPC disrupts CaM binding to and regulation of ryanodine receptor type 1 (Ryr1), Inositol 1,4,5-trisphosphate receptor type 1 and the plasma membrane calcium pump inhibiting CaM-mediated calcium regulation [64].

## 9. Discussion

**Figure 4:**
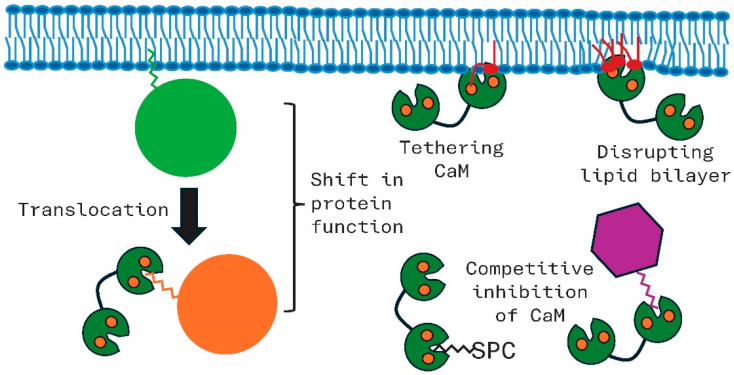
Functions of calmodulin (CaM) mediated by lipid binding. CaM binding to the prenyl group of membrane-bound proteins causes their release and translocation into the cytoplasm. This shift in membrane association versus cytosolic also leads to a shift in function of the prenylated protein. The binding of CaM to phospholipids can both tether it to the membrane as well as disrupt the lipid bilayer. In the cytosol, prenylated protein or sphingosylphosphorylcholine (SPC) binding to CaM acts as a competitive inhibitor interfering with the regulatory proteins’ ability to bind to other protein targets. The images are meant to convey the events but not details about the binding interactions between CaM and its lipid targets which are covered in the main text.

Calcium-bound CaM binds to lipids, leading to various scenarios as summarized in Figure 4. Binding to the lipid tail of prenylated small G proteins has been well studied for RalA and KRAS4b. This interaction leads to the translocation of the membrane-bound prenylated protein into the cytoplasm. Coupled with this, the shift from the membrane-bound CaM-free state to a cytosolic Ca^2+^–CaM condition is linked to different functions carried out by the prenylated target. The binding of CaM to prenylated proteins or to free lipids (e.g., sphingolipids; not shown in Figure 4) also competitively inhibits the ability of CaM to bind to and regulate other CaMBPs [64]. Ca^2+^–CaM binds to phospholipids in biological membranes. Binding to PS and PE has been well studied. This binding can lead to tethering of CaM to the membrane and/or the disruption of the lipid bilayer. As detailed in Scollo and colleagues’ work, CaM interactions with phospholipids leads to altered membrane properties, including destabilization and altered fluidity [56].

The impact of CaM–lipid binding is different for different targets. While various Ras GTPases (e.g., KRAS4b, RalA, Rac1) and other proteins (e.g., Cdc42) have prenyl groups that link them to membranes, their regulation involves different mechanisms. For example, while RalA and KRAS4b are both dislodged from the membrane by Ca^2+^–CaM binding, binding is not involved in Rac1 dislocation and there is no evidence Ca^2+^–CaM binding affects the lipid-mediated membrane binding of G beta subunits or nuclear lamins.

### 9.1. Binding Efficiency of Lipids to Calmodulin

Lipid binding to CaM is calcium-dependent. It involves hydrophobic pockets in regulatory protein’s dual lobes that open when it is charged with calcium. As seen above, the prenyl group of RalA binds to the hydrophobic pocket of the N-lobe of CaM, while the lipid tale of KRAS4b binds to the C-lobe. The significance of this differential lobe binding has not been addressed. It could be due to the differential binding of calcium to its C-lobe and N-lobe, where the C-lobe affinity is 10 times that of the N-lobe since KRAS4b binds the C-lobe preferentially [10,46]. While RalA binding via the N-lobe might seem to contradict this idea, the C-lobe is not available for binding because it is already bound to a canonical calcium-dependent binding domain [53]. Binding may also relate to the different length or types of prenyl groups, since RalA has a 20-carbon geranylgeranyl tail while KRAS4b has a 15-carbon farnesyl group bound to it. Some insight into these issues has been revealed.

A dissociation constant (Kd) is a measure of binding affinity between two molecules, such that the lower the Kd value, the stronger the binding [66]. The lack of extensive, comparative and consistent studies on estimating Kd values for CaM–lipid binding limits a detailed comparison [67]. However, a summary of some of the work done on binding of prenylated proteins to CaM is worth introducing. For example, Nef affinity for Ca^2+^–CaM was evaluated using surface plasmon resonance on a BIAcore instrument, and different experiments revealed two independent dissociation reactions for Nef/CaM with Kds of 3.74 × 10^−9^ and 9.36 × 10^−7^, supporting a high binding affinity [20,22]. As with Nef, the same methodologies revealed a Kd for myristoylated CAP-23/NAP-22 binding to CaM that ranged from 1.37 × 10^−8^ to 8.75 × 10^−8^ [22]. Using surface plasmon resonance, a more soluble farnesyl-modified RalA than its normal geranylgeranyl moity was used to estimate prenylated RalA binding to CaM [53]. This prenylated RalA revealed a Kd of 19 ± 3 nM. While multiple factors come into play in assessing Kd, these results provide some insight into the high affinity that exists for CaM binding to protein-bound lipid moieties.

That data might suggest that related CaM binding of free lipids could also show similar affinities. Existing research supports this idea, but only under certain conditions. The binding of SPC to CaM is stronger when SPC is in micelles involving submicromolar dissociation constants that range widely (0.15–0.003 mM to 15–30 nM) depending on micelle concentrations and experimental conditions [64]. How this relates to the ability of SPC to inhibit CaM function by dislodging previously bound proteins remains to be resolved, but phospholipid binding to CaM often appears to occur in the nanomolar range, while CaM binding to protein targets typically involves micromolar dissociation constants, indicating more research is required [63,68]. With that in mind, it is important to see what is known about the Ca^2+^–CaM–lipid binding mechanism.

### 9.2. Prenyl Group–Calmodulin Binding Mechanism

**Figure 5:**
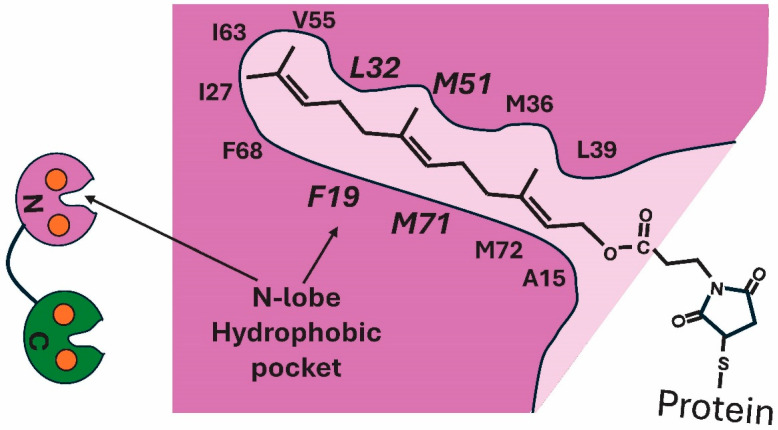
Farnesyl lipid moiety inserted into the hydrophobic pocket of the N-lobe of calcium-bound calmodulin. Hydrophobic amino acids of the binding pocket are shown. The larger, italized hydrophobic residues (F19, L32, M51, M71) are those involved in Ca^2+^–CaM protein binding. Modified from Figure 3D and data in [53].

NMR structural analysis coupled with nuclear Overhauser effect spectroscopy (NOESY) experiments revealed the first structure of CaM binding to a lipidated GTPase [53]. Chamberlain and colleagues used a shortened soluble farnesyl group to gain insight into lipid moiety binding of RalA to CaM [53]. The work revealed that the farnesyl group interacts with the “hydrophobic aliphatic and aromatic sidechains of residues” in the hydrophobic pocket of the N-lobe of Ca^2+^–CaM. Prenyl groups are highly hydrophobic lipid anchors that interact with hydrophobic amino acids. Their binding to FLMM amino acids in the hydrophobic pockets of the N- and C-lobe of Ca^2+^–CaM is primarily mediated by hydrophobic interactions that exclude water, allowing other binding interactions to come into play (e.g., van der Walls, Pi-stacking) [69]. The length of the prenyl group markedly increases binding since longer chains increase hydrophobicity, leading to deeper membrane insertion, as well as affecting hydrophobic pocket binding. Thus, it would be expected that RalA would show tighter binding than KRAS4b. It has been proposed that a Lys/Arg-rich polybasic amino acid adjacent to the lipidated C- or N-terminus plays a part in binding, an event that is sensitive to phosphorylation. Figure 5 presents a figure of farnesyl binding to the N-lobe of farnesyl–RalA. At the base of the hydrophobic pocket of CaM’s N-lobe, the lipid moiety interacts with Ile27, Leu32N/Leu105, and Ile63N [53]. Within the pocket, Phe19 and Phe68 interact with the prenyl anchor, as do Met36, Met51, Met71 and Met72N at its entrance. While this explains how the experimentally introduced farnesyl group fits into the pocket, it does not fully reveal the interaction or which of the pocket residues are essential. For Ca^2+^–CaM protein target binding, the FLMM amino acids in the hydrophobic pocket mediate binding via F19, L32, M51 and M71 in the N-lobe, and F92, L105, M124 and M144 in the C-lobe binding pockets [8,9]. Are the same FLMM residues critical for lipid group binding also critical in binding to protein motifs? Also, how does this explain the weaker binding characteristic of different lipid groups. For example, the length of the prenyl group matters, since longer chains increase hydrophobicity, leading to deeper membrane insertion as well as affecting hydrophobic pocket binding [47,53,70]. Are the same pocket residues that bind to the N-term (e.g., RalA) versus the C-term (KRAS4a) involved in binding between lipids. Understanding this may assist in clarifying the binding efficiency between certain lipidated proteins and CaM.

The length of the prenyl group markedly increases binding since the longer chains increase hydrophobicity, leading to deeper membrane insertion as well as affecting hydrophobic pocket binding. Thus, it would be expected that RalA would show tighter binding than KRAS4b. It has been proposed that a Lys/Arg-rich polybasic amino acid adjacent to the lipidated C- or N-terminus plays a part in binding, an event that is sensitive to phosphorylation [66].

The development of therapeutics that target prenylated proteins has been a focus, because protein prenylation is linked to the pathogenesis of a diversity of diseases, including cancers and cardiovascular diseases [71,72]. The question is whether a greater understanding of calcium-dependent prenyl-pocket binding will lead to the development of therapies that can control the amount of membrane versus cytosolic levels of the proteins, and hence have potential for use in treating oncogenic cells.

### 9.3. Calmodulin Interactions with the Cell Membrane

The same issues come into play in attempting to understand how PS, PL and sphingolipid binding are mediated and how this binding affects basic cell functions. But it is possible to speculate on this based on the data that exists. Studies on membrane phospholipid binding indicate that the interaction between Ca^2+^–CaM and its lipid targets is not a straightforward event but involves several steps [47,56]. The initial event is the charge-dependent association of Ca^2+^–CaM with a bilayer via electrostatic interactions that are mediated by calcium ions. Called “calcium-bridging”, this initial interaction sets the stage for hydrogen bonding between the regulatory protein and certain phospholipids, such as PE, leading to disruption of the lipid bilayer. The disruption exposes hydrophobic lipid chains, making them available for binding to the FLMM hydrophobic pocket of Ca^2+^–CaM, which in turn leads to further disruption of the lipid bilayer. Many aspects of this sequence remain to be clarified, especially the independent or, possibly, co-operative role of PS, PE and other membrane targets of Ca^2+^–CaM. In the meantime, it sets the stage for further insight into membrane fusion, an area worth examining.

The final mechanism of bilayer fusion that is involved in fertilization, cytokinesis, endocytosis, exocytosis and other events has remained elusive over the last half century [72,73,74]. In the case of calcium-regulated exocytosis, it is generally accepted that the final fusion event involves synaptotagmin, putting the lipid bilayers in a position to facilitate their merger [39,75]. But how that fusion, the actual coalescence of the two juxtaposed lipid bilayers, occurs remains unexplained. What must occur is the organized disruption of the vesicular lipid bilayer coupled with collaborative disordering of the plasma membrane bilayer, so the two can coalesce from one continuous bilayer. Based on the ability of CaM to bind to the ever-present PS and PE at the exocytotic membrane fusion point, it is possible that Ca^2+^–CaM provides both the disruption and stability needed for bilayer fusion. Future work should take this possibility into consideration.

## 10. Conclusions

The binding of Ca^2+^–CaM to lipid molecules has multiple significant roles in biological processes, from overseeing protein localization and controlling signaling events, to potentially collaborating in biomembrane fusion. Considering its importance in the function of prenylated oncogenic proteins, it may also offer new routes for developing cancer therapeutics. The overwhelming cellular importance of CaM in controlling cell function via its binding to and regulation of over 400 CaMBPs becomes even more significant with revelations related to its ability to bind to lipids.

## Data Availability

No new data were created or analyzed in this study. Data sharing is not applicable to this article.
